# Placebo effects beyond dopamine

**DOI:** 10.1371/journal.pbio.3002812

**Published:** 2024-09-25

**Authors:** Karin B. Jensen

**Affiliations:** Department of Clinical Neuroscience, Karolinska Institutet, Stockholm, Sweden

## Abstract

The role of dopamine in reward expectancy has led to the hypothesis that it is crucial for forming treatment expectations and placebo effects. This Primer explores a new study in PLOS Biology that presents robust evidence against the causal role of dopamine in these processes.

## Teaser

The role of dopamine in reward expectancy has led to the hypothesis that it is crucial for forming treatment expectations and placebo effects. However, a new study in *PLOS Biology* presents robust evidence against the causal role of dopamine in these processes.

Placebo analgesia represents a striking example of how the brain modulates incoming sensory information from the periphery and shapes pain perception. Dopamine has been suggested to play a central role in the cognitive processes underlying placebo responses, yet the evidence has been inconclusive. A new study [1] examined the neurobiology of placebo analgesia by testing the causal role of dopamine on the formation of positive treatment expectations and associated responses to a placebo treatment. Using a novel experimental approach in healthy volunteers, including 2 opposing pharmacological modulations of dopaminergic tone, the authors conclude that dopamine had no influence on treatment expectations and subsequent placebo analgesia. The study challenges the causal role of dopamine in responding to placebo and opens the door to exploring alternative placebo mechanisms.

Placebo effects rely on different forms of learning to create predictions of relief. In its most simple form, it can be illustrated by a patient in pain who takes a placebo pill resembling their regular pain killers. Despite being inert, the familiarity of the pill and past associations to pain relief may result in less pain—demonstrating placebo analgesia.

The psychological mechanisms involved in placebo analgesia have been widely studied and include the formation of expectations via conditioning, verbal suggestions, and social observation, acting both on a conscious and nonconscious level [2]. Yet, the details of how treatment expectations are translated into measurable symptom relief are not fully understood, despite being a question of profound relevance to medicine and human biology.

A seminal study by Levine and colleagues in 1978 [3] used a pharmacological challenge to test whether placebo analgesia is mediated by endogenous opioids. By administering the opioid antagonist naloxone to participants who responded to a placebo treatment, the analgesic effect was reversed, concluding that placebo analgesia is largely mediated by endogenous opioids. These findings were later validated by brain imaging studies showing activation of descending opioidergic pathways during placebo analgesia [4] similar to the effects of exogenous opioids [5]. The pioneering naloxone study and subsequent brain imaging research were central for demonstrating that the placebo effect, once seen as an intangible psychological phenomenon, could be explained neurobiologically. Yet, if the pain inhibition per se was mediated by endogenous opioids, what brain processes would reflect the initial formation of expectancies preceding placebo analgesia?

The release of dopamine has previously been linked to the formation of expectations during reward learning, particularly through reward prediction errors. This connection has made dopamine a central focus for understanding placebo analgesia, as the anticipation of symptom relief can be seen as a form of reward prediction, and the placebo effect as the result from prediction errors [6]. However, evidence for dopamine’s role in placebo analgesia has been inconclusive, often coming from small studies. On one hand, there are data suggesting increased dopaminergic neurotransmission in the striatum during anticipation of pain relief [7]. On the other hand, pharmacological blocking of dopaminergic neurotransmission in the striatum did not affect the magnitude of placebo analgesia reported by the study participants [8].

The present study by Kunkel and Asan et al. [1] not only challenges the causal role of dopamine in placebo responding but also questions the notion that placebo analgesia can be compartmentalized into distinct dopaminergic and opioidergic roles, representing the expectancy and inhibition of pain, respectively. This idea has been contested in previous smaller studies, such as [8,9], but the current study has several strengths that allow for stronger conclusions about the role of dopamine in placebo analgesia. One main strength is its randomized, double-blind, placebo-controlled design that included drugs that acted both to increase and decrease dopaminergic transmission. It might seem confusing that the authors included a placebo control in a placebo study, but it was a placebo control for the dopamine agonist/antagonist and not for the analgesic treatment. Additionally, the study tested the placebo effect on 2 separate occasions, 1 week apart, and included a large enough number of participants to provide more robust statistical power compared to previous studies on the same topic. The preregistration of the study significantly enhances the impact of the paper by ensuring transparency in the experimental and statistical methods, and ability to publish in spite of negative findings.

The results from Kunkel and Asan et al. [1] may seem disappointing, as they reject the hypothesis that dopamine shapes treatment expectations and placebo analgesia. However, these findings open up fresh perspectives on placebo effects beyond dopamine, leading to new testable hypotheses. Predicting placebo responses is notoriously difficult, and this well-controlled study confirms that the magnitude of an individual’s treatment expectations prior to treatment did not predict subsequent placebo analgesia. Previous studies suggest that treatment expectancy ratings correlate with placebo responses only under certain circumstances, particularly in mechanistic studies with healthy volunteers compared to clinical trials in patients with chronic pain where placebos are included as control treatment [10]. Furthermore, qualitative studies reveal that individuals suffering from chronic pain overwhelmingly deny having positive expectations due to a long history of treatment failures. Instead, they mention hope and desire for relief as concepts that better capture their reluctantly optimistic stance [11]. Patients may thus have fewer opportunities for prediction errors that adjust their pain perception to fit high expectations of relief, leading to placebo analgesia. Taken together, placebo studies in patients with chronic pain point to at least one alternate route to placebo analgesia that is related to motivation rather than explicitly reported positive expectations as there are still robust placebo responses in patients with chronic pain [11]. Instead, a two-dimensional model that includes both expectations and motivational value [expectancy*value] may better explain placebo outcomes. Despite its apparent link to reward-related functions, this model may not be as directly linked to dopaminergic transmission as reward prediction per se and may thus explain the lack of correlation to dopamine in studies using a univariate account for treatment expectations.

Considering the brain’s predictive abilities more broadly, predictions about future outcomes are made automatically and have evolved to handle uncertain and dynamic situations. In contrast, treatment expectancy is a self-reported measure that requires self-reflection. This measure likely represents one of several brain mechanisms involved in placebo analgesia and may not be directly linked to dopaminergic transmission. Placebo mechanisms that operate outside of conscious awareness, and placebo analgesia in response to open-label placebos (where patients know the treatment is inert), are examples that challenge the one-sided notion of explicit expectations and placebo analgesia. Future studies should also explore new neurotransmitters involved in placebo analgesia, such as noradrenaline, since opioids and dopamine have been the main suspects to account for placebo analgesia so far. The interplay of these (and previously unexplored) neurotransmitters and their combined influence on placebo responses offers a promising direction for research in a field with high clinical relevance.
